# A full thickness macular hole following severe preeclampsia: a case report

**DOI:** 10.5935/0004-2749.20230061

**Published:** 2023

**Authors:** Sehnaz Ozcaliskan, Ozgur Artunay, Gurkan Erdogan, Cengiz Alagoz

**Affiliations:** 1 University of Health Sciences, Beyoglu Eye Training and Research Hospital, Istanbul, Turkey.

**Keywords:** Retinal perforations, Vitrectomy, Vitreoretinal surgery, Pre-eclampsia, Tomography, optical coherence, Perfurações retinianas, Vitrectomia, Cirurgia vitreorretiniana, Pré-eclampsia, Tomografia de coerência óptica

## Abstract

We report a case of a young woman presenting with decreased vision in the right eye. One month earlier, she developed severe preeclampsia at 22 weeks of gestation and the pregnancy was terminated. Fundus examination revealed cotton wool spots and hard exudates in the macula bilaterally, with a yellow spot at the center of the fovea in the right eye. Optic coherence tomography showed a full thickness macular hole with elevated cystoid edges in the right eye. The patient was diagnosed with macular hole secondary to preeclampsia and followed up for spontaneous closure. One month after the first visit, surgical intervention was suggested due to declining vision. Three months later, the patient agreed to surgery. She underwent pars plana vitrectomy with a temporal inverted internal limiting membrane flap and C3F8 endotamponade, which provided anatomic and visual improvement.

## Abstract (pt)

Relatamos o caso de uma jovem com diminuição da visão do olho direito. Um mês antes do primeiro atendimento, a paciente desenvolveu pré-eclâmpsia grave com 22 semanas de gestação e interrompeu a gravidez. O exame de fundo revelou manchas algodonosas e exsudatos duros na mácula em ambos os olhos, com uma mancha amarela no centro da fóvea do olho direito. A tomografia de coerência óptica mostrou um buraco macular de espessura total com bordas cistoides elevadas no olho direito. A paciente foi diagnosticada com buraco macular secundário a pré-eclâmpsia e acompanhada para fechamento espontâneo. Um mês após a primeira visita, foi-*lhe* sugerida a intervenção cirúrgica, devido a um declínio em sua visão. Três meses depois, o paciente aprovou a cirurgia e foi submetida a uma vitrectomia via *pars plana* com retalho invertido de membrana limitante interna e tampão interno C3F8, o que proporcionou uma melhora anatômica e visual.

## INTRODUCTION

Macular holes are a common idiopathic occurrence in the elderly population. Other retinal pathologies may indirectly cause macular holes. For example, macular holes have been reported in association with retinal vein occlusions, diabetic retinopathy, hypertensive retinopathy, and retinal artery macroaneurysm^(1-4)^.

Preeclampsia is a serious complication of pregnancy characterized by elevated blood pressure, proteinuria, and edema. The most common ocular complication of preeclampsia is retinopathy similar to hypertensive retinopathy, with cotton wool spots, hemorrhages, edema, optic disc edema, and serous retinal detachment^(5)^. In such cases, pathogenesis is related to both retinal and choroidal ischemia secondary to arteriolar vasospasm. Elevated blood pressure leads to hypoxic injury and inflammatory cytokine release and, consequently, the inner blood-retinal barrier is disrupted and becomes more permeable. Choriocapillaris ischemia causes retinal pigment epithelium damage and subsequent impairment of the outer blood-retinal barrier. As a result, protein and fluid accumulate in the retinal layers, potentially leading to exudates, edema, and serous retinal detachment. Other retinal complications, such as Purtscher-like retinopathy, hypertensive choroidopathy, and retinal vascular occlusion, have been previously associated with preeclampsia^(6,7)^. This report describes a case of a full thickness macular hole in a woman with severe preeclampsia and then discusses its pathogenesis and management.

## CASE REPORT

A 32-year-old woman presented to our clinic with a complaint of decreased vision in her right eye. She had previously undergone pregnancy termination due to severe preeclampsia at 22 weeks of gestation. Her condition gradually improved and she was discharged on postoperative 7 day with a systemic blood pressure within the normal range. One month after termination, she noted vision loss in her right eye. Her medical history was unremarkable for any ophthalmic disease or previous trauma. The best-corrected visual acuity (BCVA) was 20/200 in her right eye and 20/32 in her left eye. Anterior segment findings were unremarkable and intraocular pressure was within the normal range in both eyes.

**Figure 1:**
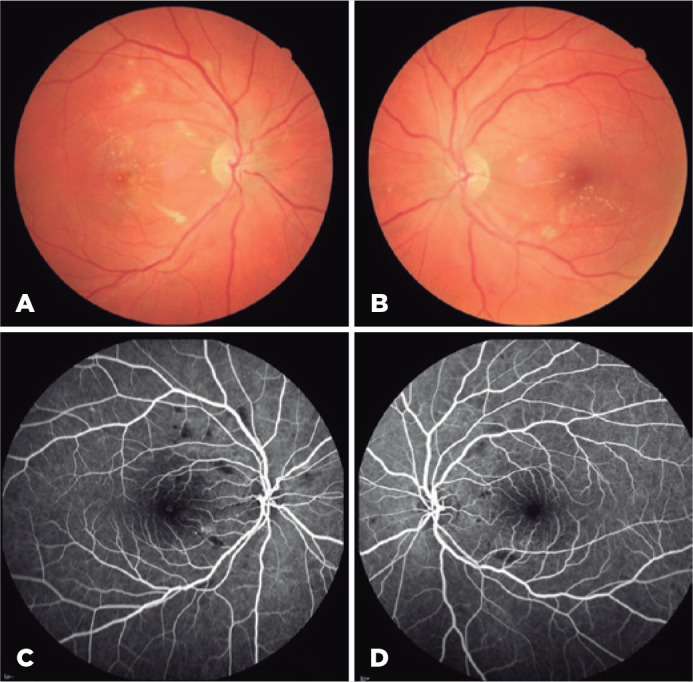
Color fundus images showing cotton wool spots and hard exudates in the macula bilaterally.eIn the right eys, a yellow spot at the center of the fovea is observed (A,B). Fluorescein angiographyeshows hypofluorescence corresponding to the locations of cotton wool spot lesions with a few pinpoint hyperfluorescent spots in the right eye (C,D).

**Figure 2:**
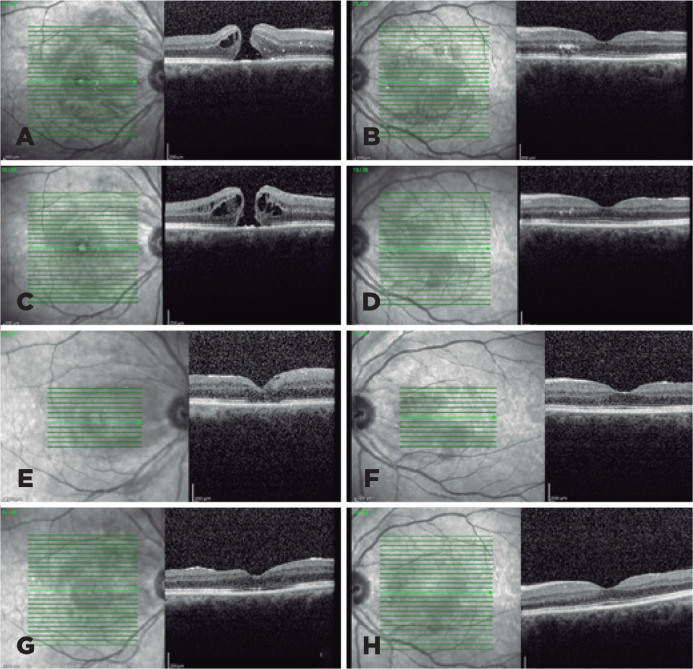
Preoperative and postoperative optical coherence tomography (OCT) scans. The baseline OCT scansgshow a full thickness macular hole with elevated cystoid edges in the right eye and hard exudates located mainly in the outer plexiform and some in the outer layer with disruption of the ellipsoid zone (EZ) in the left eye (A, B). The preoperative OCT scans revealed that the hole’s edges were thickened by intraretinal cysts, and theeEZ was recovering in the left eye (C, D).tOne montheafter surgery, OCT imaging showed closure of the hole and external limiting membrane (ELM) and disruption, while the left eye was normal (E, F). Three months after surgery, OCT imaging showing that the macular hole was closed with ELM and EZ restoration and normal foveal contour, and the left eyedshowed complete restoration of the ELM and EZ (G, H).

Fundus examination showed cotton wool spots and hard exudates in the macula bilaterally. In her right eye, a yellow spot at the center of the fovea was also observed (Figure 1, A and B). Fluorescein angiography revealed hypofluorescence in the same locations as the cotton wool spot lesions with a few pinpoint hyperfluorescent spots in the right eye. The optic disc angiogram findings were normal bilaterally (Figure 1, C and D). Optic coherence tomography (OCT) revealed a full thickness macular hole with elevated cystoid edges in the right eye and hard exudates located mainly in the outer plexiform, as well as some in the outer layer with disruption of the ellipsoid zone (EZ) in the left eye (Figure 2, A and B). All other laboratory data and systemic examination results were unremarkable, including blood pressure.

The patient was diagnosed with a macular hole secondary to preeclampsia and followed up for spontaneous closure. One month after the first visit, surgical intervention was recommended due to declining vision. The patient refused surgery but attended follow-up regularly. Three months later, the edges of the hole were thickened by intraretinal cysts, and the BCVA and EZ were recovering in the left eye (Figure 2, C and D). The patient approved the surgery and underwent pars plana vitrectomy with a temporal inverted internal limiting membrane (ILM) flap and C3F8 endotamponade. She was advised to maintain a prone position. One month after surgery, OCT imaging of the right eye revealed closure of the hole and external limiting membrane (ELM) and EZ disruption. The B-scan of the left eye was normal (Figure 2, E and F). Three months after surgery, OCT imaging showed complete closure of the macular hole with ELM and EZ restoration and normal foveal contour; OCT scans of the left eye revealed complete restoration of the ELM and EZ (Figure 2, G and H). The patient’s BCVA improved to 20/40 and 20/20 in eright and left eyes, respectively.

## DISCUSSION

This case demonstrates an unusual association of a macular hole with preeclampsia. A macular hole as a complication of preeclampsia is uncommon, and various mechanisms may be attributed to macular hole development in this case. Although the pathogenesis of macular holes remains unclear, OCT has revealed that anteroposterior and tangential traction plays a key role^(8)^. In the present case, the posterior hyaloid was attached to the macula, as shown by OCT images, and no signs of epiretinal membrane were present. Thus, traction was not associated with macular hole formation in this case.

In a previous study of a preeclamptic patient, OCT revealed macular edema with tent-shaped organization and massive neurosensorial retinal detachment, which induced elevation of the inner and outer retinal layers^(9)^. Blood-retinal barrier impairment can lead to fluid transudation and proteinaceous exudate accumulation in the subretinal space, resulting in serous retinal detachment. In most cases, the subretinal fluid di­sappears spontaneously during the postpartum period. It is known that the retina is thinnest at the foveal floor. In our patient, acute edema and elevated pressure in the subretinal space presumably provoked formation of the full thickness macular hole in the right eye. Rupture of distended Müller cells and coalescence of perifoveal cystoid spaces may also lead to foveal floor opening. Moreover, macular and choroidal ischemia with consequent retina pigment epithelium dysfunction may render the fovea vulnerable.

Alternatively, this case could be coincidental, particularly given the unilateral involvement. The retinal and choroidal ischemic changes may have promoted the progression of a previously undiagnosed hole. However, considering our patient’s medical history and clinical findings, this seems very unlikely.

Since spontaneous closure of an idiopathic macular hole is rare, surgery is often required. However, spontaneous closure has been reported in other etiologic forms of macular holes^(10)^. We initially observed the patient for spontaneous closure, but further visual impairment compelled us to recommend surgery after the first month. Due to the delay in the patient consenting to surgery, vitrectomy was performed 4 months after the initial diagnosis. Although the functional and anatomical outcomes are similar, we prefer to perform the temporal inverted ILM flap technique instead of ILM peeling to decrease the risk of iatrogenic trauma to the nasal fovea and dissociated optic nerve fiber layer appearance. In this case, we achieved visual improvement of 20/40 with a normal foveal contour. Previously, Shukla et al. reported Purtscher-like retinopathy and serous retinal detachment leading initially to a unilateral macular hole and ultimately macular tractional detachment in a preeclamptic woman^(7)^. They performed vitrectomy 4 months after diagnosis; however, they achieved only a modest vision gain. This outcome might be related to coexisting tractional macular detachment in that case.

In conclusion, this case demonstrates a rare retinal manifestation of preeclampsia. Further research is required to understand the underlying mechanisms in macular hole development. Early intervention and surgical management may provide good visual and anatomic outcomes in such cases.
